# The New Era of Therapeutic Strategies for the Management of Retinitis Pigmentosa: A Narrative Review of the Pathomolecular Mechanism for Gene Therapies

**DOI:** 10.7759/cureus.66814

**Published:** 2024-08-13

**Authors:** Praveena P Nair, Manjiri P Keskar, Pramod T Borghare, Hellen Y Dzoagbe, Tanish Kumar

**Affiliations:** 1 Otolaryngology, Mandsaur Institute of Ayurved Education and Research, Bhunyakhedi, IND; 2 Otolaryngology, Parul Institute of Ayurved, Parul University, Limda, IND; 3 Otolaryngology, Parul institute of Ayurved, Parul University, Limda, IND; 4 Otolaryngology, Mahatma Gandhi Ayurved College Hospital and Research, Wardha, IND; 5 Anatomy, Datta Meghe Institute of Higher Education and Research, Wardha, IND; 6 Medicine, Datta Meghe Institute of Higher Education and Research, Wardha, IND

**Keywords:** rhodopsin (rho) gene, genetic mutations, pathomolecular mechanisms, genome editing, stem cell transplantation, photoreceptor degeneration gene therapy, hereditary retinal degeneration, retinitis pigmentosa

## Abstract

Retinitis pigmentosa, or RP, is a group of inherited retinal degenerations involving progressive loss of photoreceptor cells- rods and cones- ultimately causing severe vision loss and blindness. RP, although a very common ailment, continues to be an incurable disease with little to be done medically. However, with the breakthroughs in gene therapy and stem cell transplantation in recent years, a new door has been opened to the treatment of RP. This narrative review summarizes the pathomolecular mechanisms of RP, focusing on the genetic and molecular abnormalities that lead to the process of retinal degeneration. In this section, we talk about the current theories of how RP develops, gene mutations, oxidative stress, and inflammation. We also delve into new therapeutic approaches such as gene therapy, stem cell transplantation and genome surgery, which are designed to either replace or repair the damaged photoreceptors to restore vision and ultimately enhance the life of the RP patient. Another topic covered is the obstacles and research frontiers of these revolutionary treatments. This article is intended to give a complete overview of the molecular processes of RP and the promising treatment strategies that could change the way this devastating disease is treated.

## Introduction and background

Retinitis pigmentosa (RP) is an assortment of eye conditions connected with hereditary retinal dystrophies, which are typified through steady degeneration involving retinal photoreceptors and this eventually results in a slow loss of vision [1]. It is otherwise known as rod-cone dystrophy since the rods primarily degenerate instead of cones; this condition is thought to occur in one out of every 4,000 people globally [2]. RP is classified according to mode of inheritance into autosomal dominant (adRP) making up about 15-25% of cases [3], X-linked RP, also known as x-RP comprising 10-15% of all cases, and sporadic-RP representing 40-50% of cases where inheritance pattern is not ascertained. Information on age at onset and the degree of visual loss determines severity of the condition in different modes of inheritance: while some cases are only diagnosed at the age of 50 due to its high acuity preservation, adRP is said to be most favorable as it is usually detected early and additionally because X-linked RP has a poor prognosis [4].

RP does not currently have a cure, and voretigene neparvovec is only available to patients who have tested positive for retinoid isomerohydrolase (RPE65) mutations [1]. The restricted therapy landscape consists of retinoids, vitamin A supplements, sunlight protection, visual aids and treatments for eye diseases aimed only at reducing the rate of disease progression [5]. The finding of new molecule drugs that target only certain receptors or paths will be the groundwork for future drug production. This article is focused on examining various therapeutic techniques and the current practice of handling RP by emphasizing the molecular path modifications associated with its progression. The review will consider pertinent aspects relating to endoplasmic reticulum stress pathways, apoptosis, redox homeostasis preservation as well as genomic stability maintenance. Besides this, gene transfer and cell manipulation are among those under investigation as possible solutions to the problem [6].

RP is a diverse cluster of hereditary degenerations that are marked by the slow deterioration of rods before cones in the eyes. This disease is widespread among people from different parts of the world and causes impairment in a lot of them. The prevalence rate for the typical form of RP -futuristic RP, not linked with any another system ailment-, is around one out of 4000 across the globe. People who are diagnosed with normal RP usually start having symptoms in the early phases of their lives with common signs involving failure to see clearly after dark, alongside progressive decline in peripheral field quality for both eyes, loss of central vision and then ultimately complete loss of sight [7]. RP is a variety of inherited retinal conditions distinguished by progressive retinal photoreceptor degeneration which results in severe vision loss and potential blindness. The disease usually starts at early or advanced ages with its features including weak night vision (nyctalopia) and narrowing of the side vision (visual field loss). The exact causes of RP remain largely unknown and there are currently no approved treatments [8]. Recent studies have examined possible remedies for RP. In tests with animal models of retrogressive performance, gene therapy has proved to be a potential lenitive measure for the blind in delaying its progression or even for restoration of sight [7]. It has been shown in animal models of RP that controlled delivery of tauroursodeoxycholic acid from biodegradable microspheres slows retinal degeneration and vision loss [9]. Treatment options with stem cell therapy for RP are also under investigation and therefore potential changes in the future seem possible [10]. Recent developments have revealed further information on the use of valproic acid orally, vitamin A as well as fish oil in the treatment of RP [11,12]. However, much research remains to be done to fully integrate knowledge about RP into clinical practice and treatment. The exact mechanisms of development and the causes of RP are still a question; moreover, positive treatment methods for this disease are also absent. For RP, future studies must be geared towards enhancing knowledge about the disease progression, identifying more efficient and specific therapies for this condition and increasing the efficiency of the examination techniques. Further, evaluating the effect of RP on the patients' quality of life (QOL) and psychological status may contribute to the all-sided treatment for the disease [13].

## Review

Pathomolecular mechanisms of retinitis pigmentosa

In 1990, the rhodopsin gene (RHO) was discovered, and it codes for Rhodopsin, a transmembrane protein that acts as a G protein-coupled receptor [14]. RHO, a protein and a chromophore called 11-cis-retinal, are found on the discs of the outer segment of rods. When exposed to light, it triggers a series of reactions in phototransduction, closing cation channels and initiating the electrical pathway. Mutations in the RHO gene are the main reason for autosomal dominant retinitis pigmentosa (adRP), making up around 25-30% of cases [15]. The most frequently studied mutation is P23H, which involves the substitution of proline with histidine at position 23 of the protein sequence. This mutation alters a protein's function, causing it to fold incorrectly and become trapped in the endoplasmic reticulum (ER). This leads to stress in the ER and ultimately results in the death of the cell [16]. The disorder’s diversity is determined by the many genes with which it is associated, along with various mutations that affect one gene [17].

Main genes involved in RP

RP is a genetically complex disease caused by mutations in more than 40 genes and the majority of those genes are involved in the photoreceptors or retinal pigment epithelium [18]. Knowledge regarding the inheritance, genes, and mutations of this disease is important for the Increase and understanding of the RP mechanism and for the development of its therapeutic approaches.

Ferrari et al. (2011) give detailed information on the genes contributing to the formation of RP as well as the mechanisms through which the mutations in the genes contribute to the degeneration of the retina. The state of knowledge on the topic of RP and the genetic basis of RP were presented, giving information about the different genes and their relation to the disease-causing mutations. The authors emphasized that the comprehensive search for the genetic defects in RP has to continue in order to map out all the genes that can cause RP and to understand how the gene mutations cause this disease to affect the retina [19].

Xu et al. focused on 60 known causative genes with the use of exome sequencing in 157 families with RP. The results showed that there are numerous types of mutations that lead to the disease, which elaborates the genericization of RP. In the light of this work, the study brought into focus the necessity of being aware of the multigenic nature of RP, in genetic testing and genetic diagnosis of RP and, the need to further search for other disease-relevant genes [20].

Even today, there remains a number of issues in understanding RP that should be researched further because, even with the advancements in discovering disease genes for RP, there are still quite a number of relevant questions that have not been answered. For instance, Kohno et al. (2013) revealed that photoreceptor proteins can activate microglial through Toll-like receptor-4 in all-trans-retinal induced retinal degeneration. This study on the interchange between the immune response and genes associated with RP implies that the photoreceptor damage is exceeded by the immune activity and therefore, more research should be done concerning the relationship between mutations in genes responsible for photoreceptors and for immunity, and their roles in the development of RP [21].

Consequently, Birtel et al. (2018) explored the clinical and genetic profiles of patients with macular and cone/cone-rod dystrophy to identify the genetic relationship and phenotypic differences between different types of rod-cone dystrophy (RJD). This underlines the fact that the genetic makeup of retinal degenerative disorders (RDD) is multi-dimensional and further underlines the requirement for precise genetic characterization before diagnosing and categorizing these disorders [22].

Rhodopsin (RHO)

Rhodopsin is a G-protein coupled receptor or light-sensitive receptor involved in phototransduction and a mutation in the RHO gene can cause adRP. Transretinal electroretinography (ERG) recordings were used by Sakami et al. in order to examine the effects of P23H mutation in RHO in relation to phototransduction. Their findings revealed that P23H mutant RHO is able to initiate phototransduction, yet the rods that contain RHO with fully developed P23H mutation are roughly 17,000 times less sensitive to light than the rods belonging to wild-type RHO(+/+). Also, rods of RHO(P23H/P23H) animals exhibited a comparably accelerated photo-response profile, indicating a malfunction of the phototransduction cascade. The result of this study is important as it gives understanding in regard to functional effects of P23H mutation on RHO linked to adRP disease [23]. Choudhury et al. (2013) have on the other hand, extended the fundamental study of RHO mutations to investigate caspase-7 in activating the unfolded protein response (UPR) and tumor necrosis factor (TNF) receptor-associated factor 2-c-jun N-terminal kinase (TRAF2-JNK) apoptosis in threonine 17 to methionine (T17M) rhodopsin mice, which is a strain of adRP. This study presented the following outcomes: caspase-7 knockout reestablishes UPR and TRAF2-JNK apoptosis, preventing extreme T17M rhodopsin-caused retinal degeneration. Therefore, this study helps to reveal the molecular pathogeny of retinal degeneration in adRP and the potential sites for therapeutic intervention [24].

Together, these studies emphasize the significance of the RHO mutations in adRP progression and show the complex cascades that occur in the degeneration of the retina. Nevertheless, it would be pertinent to identify the several gaps that request further research in the present body of literature. For example, it is still unclear at what level and in what way the phototransduction in rods of RHO(P23H/P23H) becomes impaired. In addition, the relationship between multiple apoptotic signaling pathways as well as the UPR in adRP remains to be studied in the future for the purpose of finding new treatment approaches. Thus, although the examined literature is informative on the functional and molecular changes specific to RHO mutations in adRP, there are still many unanswered questions relating to the pathogenic effects of RHO mutations in adRP. One has to therefore consider that, molecular, biological and pharmacological approaches targeting the RHO pathway of the visual retina could possibly be used for the treatment of adRP in the long run [23].

Peripherin/RDS (PRPH2)

The PRPH2 gene is one of the notable genes that if mutated can lead to an autosomal dominant form of RP. Peripherin/retinal degeneration slow (RDS), mainly found in rod and cone photoreceptor cells, is crucial for maintenance of outer segment discs of photoreceptors, essential for light absorption and signal transduction. PRPH2 functions as a kind of transmembrane protein which has an important function of assembling these outer segments [25]. The different types of changes that affect the PRPH2 gene are likely to produce an abnormal peripherin 2 function concerning its folding, stability, or interactions with other proteins. These disruptions can interfere with the development of the photoreceptor outer segments thus affecting the proper working of photoreceptor cells. Some of the commonly observed genetic changes include missense mutation that affects structure and folding of protein and frameshift mutations, which lead to PRPH2 proteins being either truncated or non-functional [26,27]. Pathological changes in the structure and function of PRPH2 are associated with impaired localization, aggregation or degradation, which in turn interferes with the outer segment structure and function. It does this in a way that leads to the continuous loss of the photoreceptor cells and subsequently leads to symptoms including vision at night, peripheral vision and later central vision loss associated with retinitis pigmentosa [28].

Usherin Gene (USH2A)

Autosomal recessive Usher syndrome type 2 (USH2A) and non-syndromic RP are caused by mutations in USH2A. Usherin is a laminin-like protein that is encoded by the USH2A gene and is important for the growth and survival of retinal photoreceptors. Progressive photoreceptor degeneration caused by usherin haploinsufficiency leads to night blindness followed by central vision loss due to constricted visual fields. The absence of usherin has been shown to cause similar retinal and auditory defects in animal models as those seen in human patients with USH2A-related disorders. In addition, usherin plays a key role in photoreceptor development and function because it is involved in structural organization and maintenance of photoreceptor outer segments. Current research is being undertaken to examine novel therapeutic strategies such as antisense oligonucleotides or gene transfer for the treatment of people with different mutations within the USH2A gene. This understanding can help identify potential targets for drug development for affected individuals [29].

Eyes Shut (EYS)

Mutations arising in the EYS gene, coding for eyes shut protein, are one of the many reasons for adRP. The primary responsibility of EYS lies in retaining the structural solidity and functionality of photoreceptor cells that exist within the retina. Individuals who are afflicted with EYS-related RP typically display a non-syndromic RP phenotype, characterized by progressive vision impairment, night blindness, and restricted visual fields. Such mutations account for about 5-16% of all cases of adRP among non-Finnish Caucasians while this figure may be as high as 30.9% in the Japanese population [30]. It also acts at the photoreceptor layer where this protein is expressed in order to maintain stability at photoreceptors. The structural organization of the photoreceptor ciliary pocket involved in protein transport between inner and outer segments is maintained by EYS as demonstrated by studies conducted on zebrafish models. Next-generation sequencing leads to molecular diagnosis; low vision rehabilitation and genetic counseling are management strategies for these patients. The precise function(s) played by EYS remain doubtful but it has been established that mutations occurring within such a gene significantly contribute to non-syndromic retinitis pigmentosa inherited in an autosomal recessive manner [31].

ATP-Binding Cassette Sub-Family A Member 4 (ABCA4)

Autosomal recessive Stargardt disease and non-syndromic RP are two conditions that have been linked to mutations in the ABCA4 gene, which encodes for the ABCA4 protein. This latter protein belongs to the ATP-binding cassette (ABC) transporter superfamily and serves an important function in photoreceptor cells’ visual cycle as well as lipid transport among others. It is found primarily on the outer segment disc membranes of photoreceptive rods and cones aiding these cells in getting rid of potentially toxic, all-trans-retinal and N-retinylidene-phosphatidylethanolamine (N-Ret-PE) [32]. To avoid lipofuscin piling up in their system and thus causing damage that leads to degeneration of their photoreceptors, ABCA4 does away with these compounds from where they are produced within this fine structure called a disc membrane. It also helps keep photoreceptor cells safe by preventing any subsequent production of retinoid derivatives that are harmful [33]. Mutations crippling the ABCA4 gene bring about slight operational changes, making it impossible for any kind of movement in Stargardt disease characterized by central vision impairment or arrival of lipofuscin at retinal pigment epithelium cells. Also known as non-syndromic RP, such mutations may lead to sight loss over time [34]. For better strategies for patients with such retinal maladies, keeping in touch with things like these will be important- since it’s only then we can understand how the molecular basis behind ABCA4's function work- in order to make anything practical happen [35].

Retinoid Isomerohydrolase (RPE65)

Mutations in the retinoid isomerohydrolase gene (RPE65) are responsible for Leber congenital amaurosis (LCA) with an autosomal recessive inheritance and non-syndromic RP. Crucial in regenerating visual pigments and essential for both cone-mediated and rod-mediated visions, retinal pigment epithelium expresses RPE65 protein. RPE65 acts like an isomer of isomerohydrolase all-trans-retinyl esters to 11-cis-retinol which is a vital step in the visual cycle, enabling phototransduction. The mutations within the RPE65 gene result in considerable loss of protein activity leading to build-up of harmful by-products that interrupt the visual cycle, causing profound visual disability from birth or early childhood. About 6% to 16% of LCA cases are linked to mutations in RPE65 while approximately 2% of adRP cases can also be traced back to this gene variant. Recent advances in gene therapies directed at RPE65 have exhibited promising outcomes thereby providing hope for successful management options for patients suffering from these types of retinal disorders [36,37].

Crumbs Homolog 1 (CRB1)

Autosomal recessive non-syndromic RP and LCA have also been linked with mutations in the CRB1 gene. The CRB1 protein is an integral part of the Crumbs protein complex required for photoreceptor cell structural integrity and functionality in the retina [38]. This protein is crucial in photoreceptor morphogenesis, adhesion between cells, and cell polarity, which are necessary for organizing retinal architecture correctly. Seven percent to 17% of LCA cases are caused by mutations in the CRB1 gene while it accounts for between 3% and 9% of RP cases. Disruption to normal development of photoreceptors occurs as these mutations often lead to either non-functional or truncated CRB1 protein, resulting in the severest form of visual impairment from childhood. For developing possible therapeutic strategies for these eye diseases, it is important to understand how CRB1 works and what pathways it interacts with [39]. Table 1 shows the genes and their functions.

**Table 1:** Genetic Disorders: Genes and Their Functions RP: Retinitis pigmentosa, adRP: autosomal dominant RP

Gene	Inheritance Pattern	Protein	Function	Associated Conditions	References
RHO	Autosomal Dominant	Rhodopsin	Light-sensitive receptor involved in phototransduction	adRP	[23]
RPGR	X-Linked	RPGR Protein	Involved in photoreceptor function and maintenance	X-linked RP	[40,41]
USH2A	Autosomal Recessive	Usherin	Role in photoreceptor development and function	Usher syndrome, non-syndromic RP	[42,43]
EYS	Autosomal Recessive	Eyes Shut	Important for photoreceptor structure and function	Non-syndromic RP	[30,44,45]
ABCA4	Autosomal Recessive	ABCA4 Protein	Involved in the visual cycle and lipid transport	Stargardt disease, non-syndromic RP	[46,47]
RPE65	Autosomal Recessive	RPE65	Critical for the regeneration of visual pigments	Leber congenital amaurosis, non-syndromic RP	[36,37]
NRL	Autosomal Recessive	Neural Retina Leucine Zipper	Transcription factor for rod photoreceptor development	Transcription factor for rod photoreceptor development	[48,49]
PRPH2	Autosomal Dominant	Peripherin	Structural protein in photoreceptor outer segments	adRP, macular degeneration	[50,51]
CRB1	Autosomal Recessive	Crumbs Homolog 1	Important for maintaining photoreceptor structure	Non-syndromic RP, Leber congenital amaurosis	[52,53]
RP1	Autosomal Dominant	RP1 Protein	Involved in photoreceptor structure and function	adRP	[54,55]

Cell-based therapies for retinitis pigmentosa

**Figure 1:**
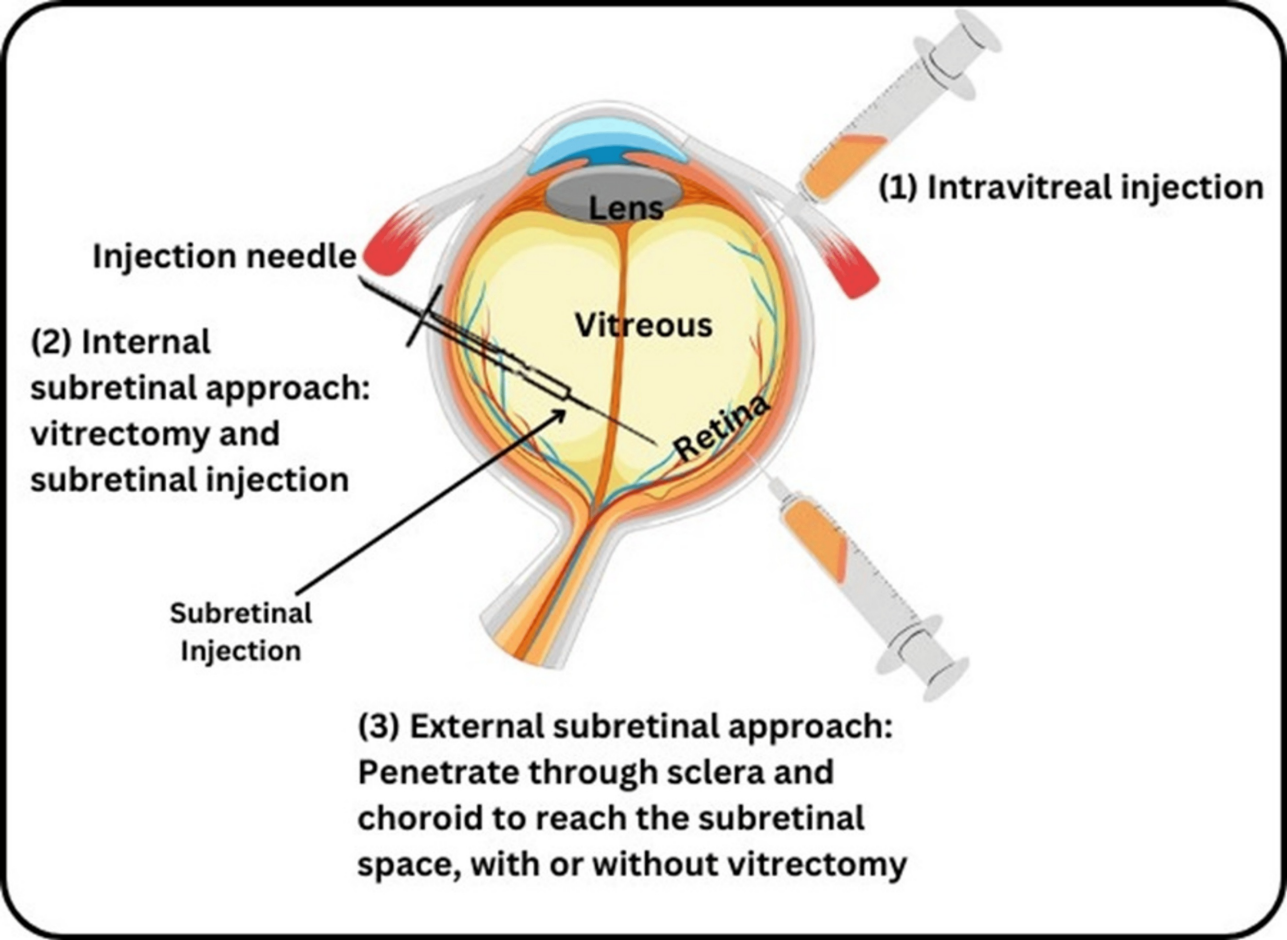
Shows stem cell treatment for retinitis pigmentosa This figure was created by Hellen Y. Dzoagbe and Tanish Kumar

RP is an inherited degeneration of the retina which is accompanied by progressive loss of photoreceptor cells causing blindness. A number of treatment modalities exist but cell-based therapy- particularly gene therapy- has come up as a promising measure to palliate this disabling condition [6]. The study of the pathogenesis of RP is discussed as a combination of genetic mutations in the genes affecting photoreceptors, oxidative stress, and inflammation. Present-day treatments of retinal diseases are mainly prophylactic in that, they seek to reduce the rate of progression and deal with the symptoms of the disease and this is still by and large the case even with researchers working on developing cell-based therapies to present several viable potentials in this area. Cell therapies target to provide replacement cells for the lost ones, to support the existing cells, and to recover the function of retinal cells in different ways. This section summarizes all sorts of cell-based therapy under consideration for RP namely, retinal cell transplant, RPE transplantation and stem cell therapy (Figure 1).

Many studies on the therapeutic effects of distinct types of retinal cells in various animal models of retinal degeneration have been published. According to Gonzalez-Cordero and colleagues (2013), therapeutic effects have been demonstrated by RPE patches produced from mutation-free pluripotent stem cells in rats and pigs. This investigation pointed out the possibility of these patches re-establishing certain qualitative function of the retina and thus opening up the possibility of future therapies focused on combating retinal degenerative diseases. The researchers tried to avoid such complications, which could appear in the case of using patient-derived cells, which makes the treatment more effective and safer by using mutation-free pluripotent stem cells. The outcomes of these experiments therefore provide a basis for the clinical use of these findings in human patients with retinal degenerative diseases, thus establishing a potential for creating new therapeutic interventions that may have a valuable impact on patients’ vision [56]. In the same way, Sharma et al. (2019) discovered that rodent and pig models with retinal degeneration were rescued by clinically tested RPE patches from stem cells. This shows that RPE cell-based therapies have potential for treating retinal degenerative diseases, given the pre-clinical evidence of the use of induced pluripotent stem cell-derived retinal pigment epithelium (iPSC-RPE) patches for the treatment of retinal degenerative diseases. The use of stem cell-based cell replacement therapy aims to replace those photoreceptors that are lost or not functioning through the application of clinical-grade RPE cells derived from stem cells. Nevertheless, the presented studies can be seen as the first steps toward the generation of fresh tissues and the development of regenerative therapies for retinal diseases [57].

Integration and Maturation of Transplanted Cells

The outer retinal degeneration also has a component in the effectiveness of cell integration with the stage considered as critical. Non-surgical intervention gave better results and the murine models used for studying inherited photoreceptor degeneration included the following factors: The integrity of outer limiting membrane and gliosis or scarring in the retina. For instance, inner limiting membrane; integration is likely to be high when this sort of limiting membrane is intact; gliosis on the other hand is likely to be low in models that have little integration. This appears to indicate that there is a need to preserve the retinal milieu, as a less interrupted one is more likely to entwine the grafted cells [58].

Bird et al. (2014) gave evidence for the safety and efficiency of human retinal progenitor cell (hRPC) transplantation in the degenerative model and focused on the photoreceptor’s preservation and retinal functional enhancement. Their work opens the door for subsequent investigations focusing on applying these conclusions to practice which may provide patients having severe retinal diseases with a new treatment option. In future studies related to this field, it will be crucial to value the long-term success and the role of hRPCs more profoundly and broadly [59].

Mechanisms and Molecular Pathways

There are various researches that have discussed the cellular and molecular processes by which retinal cell transplantation brings about its therapeutic impacts, especially in regard to retinal degeneration. Sennlaub et al. (2013) showed that intravitreally injected dental pulp stem cells increased the retinal ganglion cell (RGC) survival and axon regrowth of axotomized RGCs. This was said to have been due to neurotrophic factors that support the health and regeneration of neurons and suggested neurotrophic signaling pathway as one of the essential mechanisms in positive stem cell-derived therapies in the treatment of diseases affecting the retina [60].

Also, Schwartz et al. (2016) described the morphological alterations underlying the progression of advanced age-related macular degeneration (AMD) particularly, the loss of the RPE and photoreceptors in geographic atrophy (GA) using postmortem human eyes donated in Ohio. Based on their results, they have identified some potential candidates for cell-based therapies in the hope of reviving the function of RPE and nourishing photoreceptors. Thus, the study of molecular events at this interface can help in tailoring the approaches that target the degeneration process, providing the tools that improve the efficiency of cell-based therapies in retinal disorders. Collectively, these works highlight neurotrophic factors and the cellular contextual support in translating the beneficial aspect of retinal cell transplants for treating retinal degeneration diseases and indicate a way for further research in enhancing these goggle strategies [61].

Managing retinitis pigmentosa: treatment options and lifestyle changes

**Figure 2:**
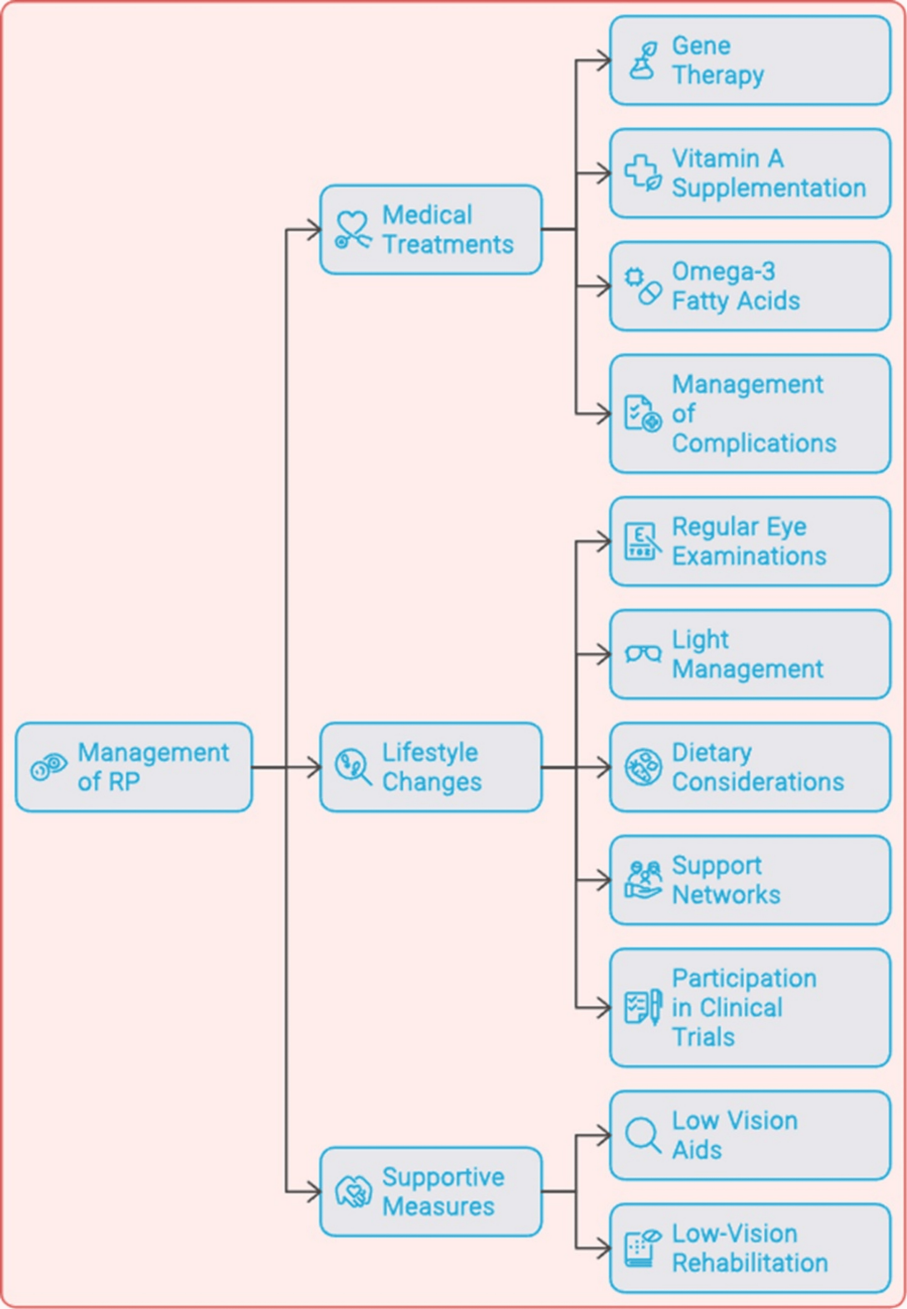
Managing retinitis pigmentosa (RP) This figure was created by Hellen Y. Dzoagbe and  Priti Karadbhajne

In managing people with RP, there will be a need to use different approaches. This includes combining various treatment options and lifestyle modifications with an aim of preserving vision and improving the quality of life for affected individuals. Although RP is not curable at present, there are several treatment modalities that provide hope for slowing down disease progression and may even help regain visual function. Figure 2 illustrates managing RP.

Gene therapy is considered one of the most promising innovations in RP treatment. Voretigene neparvovec-ryzl (Luxturna®) has been green-lighted by FDA for patients suffering from biallelic RPE65 mutation-associated retinal dystrophy [6]. Retina can be restored to respond to light and improve eyesight, especially in dim light with a healthy copy of the RPE65 gene directly injected into it.

Treatment options

Gene Therapy

The advances made in the field of gene therapy provide significant effectiveness in the treatment of RP especially taking the phase I study by Ghazi et al. (2016) into consideration. This study was done only on patients with RP caused by mutations in the c-mer proto-oncogene tyrosine kinase (MERTK) gene, which codes for protein involved in the phagocytosis of photoreceptor outer segments by RPE cells. Their phase II randomized dose-ranging trial included the intraocular subretinal injection of an adeno-associated virus (AAV) vector containing the human MERTK gene to the retina of affected patients. From this trial, it was evident that this type of gene therapy was safe and had no side effects on individuals undergoing the process. Further, there were some indications of positive effectiveness, such as patients’ improvement in visual function after the application of the analyzed treatment. This is especially notable bearing in mind that RP is a progressive disease characterized by incremental/gradual loss of photoreceptor cells leading to complete loss of vision and blindness. Nevertheless, the authors underlined the necessity of further investigations in order to compare the long-term washout effects of the therapy and to test its efficacy in other genetic subtypes of RP. RP is another recessive disorder marked by significant genetic heterogeneity and over 70% of reported genetic alterations are involved in the disease progression. Further research will be required to establish whether the same strategy of gene therapy can also be effective in other forms of RP attributable to other genes [62].

Stem Cell Transplantation

RP can result in severe loss of vision or total blindness. There is as yet no known treatment for RP, although the use of stem cell transplant has been identified as a possible means of dealing with the disease and even helping to regain vision [63].

Retinal progenitor cells (RPCs), mesenchymal stem cells (MSCs), and induced pluripotent stem cells (iPSCs) are being researched for application to RP treatment. RPCs have the ability to possibly differentiate into a number of retinal cell types such as the photoreceptors and a number of agendas have shown positive results pre-clinically. MSCs that are obtained from the patient’s own bone marrow or adipose tissue can release neurotrophic factors such as brain-derived neurotrophic factor (BDNF) and glial cell line-derived neurotrophic factor (GDNF) that preserve the structure of retinal cells and reduce inflammation. The iPSCs can be derived directly from the patient’s cells, then can be directed to differentiate into photoreceptor-like cells and transplanted to the regions of the degenerating photoreceptors in the retina [64]. Stem cell transplantation trials for RP have provided evidence of its safety and effectiveness, and clearly, the condition of some patients who underwent the procedure reported amelioration in their sight and the physical state of their retinas. However, some issues still exist, for instance, scarcity of ideal donor tissues, immune rejection and the potentiality of surgical complications [65].

Santos-Ferreira et al. (2015) conducted a study investigating the regenerative capacity of cell transplants in gaining back daylight vision in animal models of retinal degeneration. Their work proved that cell transplantation may restore visual capabilities of patients, creating the basis for stem cell therapy of RP and similar diseases. The researchers grafted hRPCs that were differentiated from mouse embryonic stem cells (mESC) into the retina of RP mouse models. They also discovered that the cells that were transplanted, had a capacity of differentiating into fully developed photoreceptive cells in the host retina. In another important aspect, it was established that this cell transplantation caused considerably enhanced abilities to see, particularly in daylight. This study evidenced that stem cell-based therapies are viable means for treating RP and other retinal degenerative diseases in hope of vision recovery. Compared to cell transplantation, which involves restoring lost or damaged photoreceptors with healthy functional cells, cell transplantation may help stop or even reverse the deterioration of a person’s vision. Also, the capacity to regain daylight vision can be considered as a major advantage, as many of the patients suffering from RP face problems with light adaptation and brightness. As far as further studies are concerned, more will be required to fine-tune this strategy and, in particular, to investigate its applicability to clinical trials in humans. The study by Santos-Ferreira et al. can be stated to be a step forward towards the development of regenerative medicine for treatment of the retinal diseases. With the development of stem cells as one of the approaches to replace the irreversibly damaged tissues of the retina, the scientific community activates the efforts to improve the condition of the persons, suffering from these diseases and to deliver the ultimate gift - the vision back [66].

Genome Surgery

Tsai et al. conducted a research study and examined the possibility of applying clustered regularly interspaced short palindromic repeats (CRISPR) based genome surgery in the case of adRP. The study indicated encouraging results, which makes it possible to consider the possibility of using the method of genome surgery for the correction of genetic mutations in RP. Thus, adRP is a form of the disease that is characterized by a single gene mutation inherited from one parent. Tsai et al. looked into the feasibility of applying CRISPR for directly altering these mutations in specific photoreceptor cells. Thus, to treat inherited blindness, the researchers attempted to deliver the CRISPR components to the retina directly, in order to allow for the gene function to return to normal and thus save the photoreceptors from degeneration [67].

By their study, Tsai et al. illustrated that CRISPR-genome surgery could work in the treatment of mutations in adRP animal models. It demonstrated that the therapy might help to slow down the degradation of vision and even protect the retina. Therefore, these findings lay the basis for the idea that in adRP, the CRISPR/Cas system might act as a potential therapy. But the authors also rightly pointed out the drawbacks and imperfections of this kind of technology. Off-target consequences, the question of how to deliver the vectors to specific retinal cells, and the question of immune responses are more problems that need to be solved. Nevertheless, the work of Tsai et al. can be considered significant progress in the sphere of potentials for the application of genome surgery to the treatment of RP. Today, using the technique of gene editing, scientists are striving for more individualized treatment that would target the genetic prerequisites of the disease. Thus, the development of such therapeutic approaches as genome surgery can be considered a hope for vision restoration and significant quality of life enhancement for people with this severe pathology [67].

Challenges and future directions

To increase safety and efficacy, certain issues need to be focused on in the field of gene therapy for retinal diseases. One major concern is immune reactions and vector-related toxicity, which occur when viral vectors initiate immune responses that can cause inflammation and harmful effects on the outcomes of treatment [68]. To add to this, the possibility of having unintended side effects as well as genotoxicity present a problem because sometimes therapeutic vectors can get integrated into genomes, causing harmful mutations. As such researchers should come up with means such as less immunogenic vectors among other strategies, targeted delivery systems or comprehensive preclinical testing protocols for ensuring safety. Another crucial focus area is the final step of vector delivery and the precision with which vectors target specific cells. The enhancement of the advancements in viral vector development, especially with AAV vectors’ efficiency, has improved their ability to transduce retinal cells. It is important to target specific layers and types of retina cells for high therapeutic efficacy; this can be achieved by using ligands which are cell-type specific or promoters that bind selectively to receptors on target cells. Besides, for better treatment outcomes, transduction efficiency enhancement must be achieved through optimizing the design of vectors and introducing new delivery methods like electroporation or micro-injection. Finally, gene therapy in the future may entail combining it with other therapies such as pharmacological agents or stem cell therapies. The use of personalized approaches based on the genetic and phenotypic makeup of patients can improve treatment efficacy further by addressing the complexities associated with different types of retinal disorders thus opening avenues for more effective individualized therapeutic strategies [69].

## Conclusions

RP is classified as a group of hereditary diseases affecting the retina and characterized by the gradual destruction of photoreceptor cells and, consequently, a worsening of visual acuity and night vision to complete blindness. Though a frequent complaint, RP is still an untreatable condition with very few available treatments. However, with the appearance of new methods in gene therapy, stem cell transplantation, and genome surgery, new opportunities for the treatment of RP have appeared. Based on the above discussion, this review has presented the pathomolecular aspects of RP with accentuation on the genetic and molecular defects that cause retinal dystrophy. The methods that are used as the treatment today, such as gene therapy, stem cell transplantation and empowered genome surgery have proven to be effective in halting the progress of the disease and in some instances, even reversing the consequences and regaining vision. Recently, gene therapy has shown safety and efficacy in clinical trials and generally using adeno-associated virus vectors. Transplantation has also provided hope in the alternative of dead photoreceptors with functional cells, and the general slicing and dicing of genes using the CRISPR system to correct mutations. Nonetheless, to advance the safety and efficacy of these therapeutic approaches, there are several issues, and limitations that require solving. These are immune-mediated toxicity, vector-based toxicity and other toxicities, off-target effects and the need to have efficient and specific delivery systems. However, the advancements noted in the conducted studies on RP indicate a possibility of finding an efficient treatment for this debilitating disease. The future research should target making these therapeutic practices more effective and efficient by the combination of treatment options or tailoring of treatment according to the specific patients.
